# Effect of coagulation function on cerebral microbleeds in intracerebral hemorrhage

**DOI:** 10.1002/brb3.1634

**Published:** 2020-04-19

**Authors:** Hui Zhang, Jian Deng, Nianlong Sun, Liangyu Zou, Jing Han, Chen Wei, Yitao He

**Affiliations:** ^1^ Department of Neurology Shenzhen People’s Hospital The First Affiliated Hospital of Southern University of Science and Technology The Second Clinical Medical College of Jinan University Shenzhen Guangdong China; ^2^ Department of Radiology Shenzhen Bao‐An People’s Hospital Shenzhen Guangdong China

**Keywords:** activated partial thromboplastin time, cerebral microbleeds, coagulation function, intracerebral hemorrhage

## Abstract

**Objective:**

Our study aimed to confirm whether coagulation function of patients presenting with intracerebral hemorrhage (ICH) was associated with onset of cerebral microbleeds (CMBs).

**Methods:**

A total of 174 patients with basal ganglia ICH were divided into CMBs and non‐CMBs groups. Indicators of coagulation function and other clinical data that included fibrinogen (FBI), prothrombin time (PT), activated partial thromboplastin time (APTT), and the international normalized ratio (INR) were compared by univariate and multivariate analysis between the two groups. A receiver operating characteristic (ROC) curve was plotted to determine the predictive value of coagulation function indicators for CMBs.

**Results:**

Univariate analysis showed that APTT levels was significantly higher in the CMBs group than the non‐CMBs group (30.20 ± 5.18 vs. 27.95 ± 4.19; *p* = .004), while there was no significant difference between PT, INR, and FBI. The proportion of male patients in the CMBs group was significantly higher than the non‐CMBs group (76.58% vs. 52.38%, *p* = .001). Multifactor logistic regression analysis demonstrated that APTT and male gender were independent risk factors for CMBs in patients with ICH (OR 1.100, 95% CI: 1.026–1.180, *p* = .008; OR 2.957, 95% CI: 1.500–5.826, *p* = .002; respectively). ROC curve analysis indicated that the area under the curve of APTT and male gender for CMBs in patients with ICH was 0.641 and 0.621, respectively (*p* = .002 and .008; respectively).

**Conclusion:**

APTT was an independent risk factor for CMBs in patients with ICH.

## Graphical abstract

(a) Activated partial thromboplastin time was an independent risk factor for CMBs in patients with intracerebral hemorrhage (ICH); (b) international normalized ratio and fibrinogen were not independent risk factor for CMBs in patients with ICH.

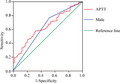

## 1 INTRODUCTION

Cerebral microbleeds (CMBs) are low‐density lesions with a diameter of 2–10 mm, which are round or oval in shape, without edema around the lesion that are discovered by T2‐weighted imaging or susceptibility‐weighted imaging (SWI) (Renard, Tatu, & Thouvenot, 2018). Histopathology showed that deposition of hemosiderin is associated with blood leakage from tiny blood vessels in the brain (Liu et al., 2017). The prevalence of CMBs in patients with spontaneous intracerebral hemorrhage (ICH) is as high as 60% (Wilson et al., 2016), and CMBs are correlated with both the occurrence and recurrence of ICH positively (Charidimou, Imaizumi, & Moulin, 2017; Lee, Kim, & Roh, 2006). In addition, patients that receive anticoagulants, antiplatelet, or thrombolytic therapy might exhibit an increase in CMBs, which further expands the incidence of ICH (Liang, Song, & Jiao, 2018).

In recent years, some researchers have repeatedly suggested that patients taking warfarin therapy or new anticoagulants would lead to an increase of CMBs (Liang et al., 2018; Orken et al., 2013; Saito, Kawamura, & Sato, 2015). In addition, patients receiving anticoagulant therapy had an eightfold higher risk of CMBs than patients not receiving anticoagulant therapy (Liang et al., 2018). At present, the mechanism on the association between anticoagulant therapy and CMBs remains unclear. Via the above studies, we speculated that altered coagulation function might be associated with the occurrence of CMBs.

However, there is no relevant research that provides the required targeted evidence, especially in the context of patients presenting with ICH. Thus, this study seeks to verify whether coagulation function is indeed associated with the occurrence of CMBs in patients with ICH.

## 2 MATERIAL AND METHODS

### 2.1 Patients

Inclusion criteria were as follows: (a) All patients met the diagnosis of acute ICH (Hemphill, Greenberg, & Anderson, 2015) and the bleeding sites were all in the basal ganglia; (b) a bleeding volume of <30 ml based on the head computerized tomography conducted at 24 hr after onset, and the calculation of bleeding volume referred to the simple formula ABC/2, which was put forward by Kothari, Brott, & Broderick (1996); (c) magnetic resonance imaging (MRI), including SWI sequence, was conducted within 7 days after admission; (d) patients had completed a study‐related blood test within 24 hr of onset; and (e) patients or their relatives signed informed consent.

Exclusion criteria were as follows: (a) Patients that were <18 years of age; (b) occurrence of secondary cerebral hemorrhages such as primary or secondary intracranial tumors, traumatic hemorrhage, and vascular malformations; (c) patients that had a history of hematological disease; (d) patients that had been administered oral anticoagulant or antiplatelet therapy for 1 month prior to admission; and (e) patients who received craniotomy.

### 2.2 Observation parameters

The following information was collected with regard to the recruited patients to this study: age, gender, history of disease (hypertension and diabetes), systolic blood pressure, diastolic blood pressure, National Institutes of Health Stroke Scale (NIHSS), platelet (PLT), creatinine (CR), urea nitrogen (BUN), fasting blood glucose (GLU), glycation hemoglobin (GHB), blood uric acid (UA), alanine aminotransferase (ALT), and aspartate aminotransferase (AST) levels.

Coagulation function detection contained the levels of fibrinogen (FBI), prothrombin time (PT), activated partial thromboplastin time (APTT), and the international normalized ratio (INR).

### 2.3 MRI judgment criteria and scanning conditions of CMBs

The diagnosis of CMBs complies with the CMBs identification criteria that were proposed by Greenberg, Vernooij, & Cordonnier (2009). Within 7 days after admission, the patient completed a 3.0 T MRI (Siemens Medical Solutions) including T1‐weighted, fluid‐attenuated inversion recovery, T2‐weighted, diffusion‐weighted MRI, and a SWI analysis. SWI scanning parameters included the following: repetition time of 28 ms, echo time of 20 ms, a field of view with dimensions of 230 × 180 mm, slice thickness of 1.2 mm, a flip angle of 15°, and a matrix of 256 × 256, a Voxel of 0.5 × 0.5 × 1.2 mm, and a scanning time of 10 min, and 43 s.

### 2.4 Ethical standard

The study was approved by the Medical Ethics Committee of Shenzhen People's Hospital. We also obtained informed consent from both patients and their families.

### 2.5 Statistical methods

Statistical analysis of the data was conducted using the SPSS version 22.0 software. Measurement data that conformed to a normal distribution were expressed as mean ± one standard deviation about the mean, and comparisons between both groups were analyzed by *t* test of independent samples. The chi‐square test was used to compare two categorical variables. Correlation analysis between CMBs and related variables, such as coagulation function, was analyzed by multivariate logistic regression analysis. A receiver operating characteristic (ROC) curve was used to evaluate independent predictive values of coagulation function and other variables on CMBs. Alpha values of *p* < .05 were considered statistical significance.

## 3 RESULTS

### 3.1 Comparison of baseline characteristics

We recruited 174 patients with ICH that met the inclusion and exclusion criteria in the Department of Neurology, Shenzhen People's Hospital between January 2014 and December 2018. Of these, there were 111 patients with CMBs (63.8%). Patients were classified into CMBs and non‐CMBs groups according to its occurrence or absence. The proportion of male patients in the CMBs group was significantly higher than that in the non‐CMBs group (76.6% vs. 52.4%, *p* = .001), and other baseline characteristics were not significant difference (All *p* > .05; as shown in Table 1).

**TABLE 1:** Baseline characteristics of the CMBs group and the non‐CMBs group

	CMBs group	Non‐CMBs group	*t* or *χ* ^2^ value	*p* value
Number of cases (*n*)	111	63		
Age (years)	60.79 ± 12.04	57.16 ± 12.73	0.600^a^	.63
Male, *n* (%)	85 (76.58%)	33 (52.38%)	10.780^b^	.001
Hypertension, *n* (%)	103 (92.79%)	57 (90.48%)	0.292^b^	.589
Diabetes, *n* (%)	23 (20.72%)	17 (26.98%)	0.891^b^	.345
Systolic BP (mmHg)	168.83 ± 23.66	164.19 ± 26.28	2.063^a^	.234
Diastolic BP (mmHg)	98.60 ± 15.40	95.21 ± 15.89	0.040^a^	.173

Abbreviations: BP, blood pressure; CMBs, cerebral microbleeds.

^a^ Conduct *t* test.

^b^ Conduct chi‐square test.

### 3.2 Comparison of coagulation function and other blood parameters

The results showed that CMBs group had significant longer APTT (30.20 ± 5.18 vs. 27.95 ± 4.19; *p* = .004) as compared with non‐CMBs group. The PT, INR, and FBI trended to be longer in the CMBs group than non‐CMBs group; however, the difference was nonsignificant (*p* > .05). The NIHSS, PLT, CR, BUN, GLU, GHB, UA, ALT, and AST were analyzed without demonstrating any significant difference between CMBs and non‐CMBs groups (All *p* > .05; as shown in Table 2).

**TABLE 2:** Coagulation function and other blood indicators of the CMBs group and the non‐CMBs group

Risk factor	CMBs group	Non‐CMBs group	*t* value	*p* value
Number of cases (*n*)	111	63		
NIHSS score	5.03 ± 4.53	5.89 ± 5.36	3.531^a^	.261
PLT (10^9^/L)	227.56 ± 61.95	229.65 ± 66.18	0.070^a^	.835
PT (s)	11.80 ± 1.39	11.62 ± 4.12	0.295^a^	.676
INR	1.09 ± 0.91	1.00 ± 0.26	0.722^a^	.447
APTT (s)	30.20 ± 5.18	27.95 ± 4.19	0.867^a^	.004
TT (s)	15.79 ± 2.04	16.13 ± 3.32	3.226^a^	.406
FBI (g/L)	3.16 ± 0.80	3.02 ± 0.70	0.297^a^	.257
CR (μmol/L)	93.97 ± 52.62	94.19 ± 124.70	0.437^a^	.987
BUN (mmol/L)	5.21 ± 2.51	5.61 ± 4.54	1.829^a^	.452
ALT (U/L)	20.32 ± 10.01	23.98 ± 18.80	13.981^a^	.095
AST (U/L)	20.17 ± 7.82	20.30 ± 8.01	0.335^a^	.916
GLU (mmol/L)	6.13 ± 1.97	6.45 ± 2.06	0.916^a^	.308
GHB (%)	5.91 ± 1.05	6.93 ± 6.78	5.294^a^	.119
UA (μmol/L)	360.35 ± 126.45	334.29 ± 97.46	2.735^a^	.159

Abbreviations: ALT, alanine aminotransferase; APTT, activated partial thromboplastin time; AST, aspartate aminotransferase; BUN, urea nitrogen; CR, creatinine; FBI, fibrinogen; GHB, glycation hemoglobin; GLU, fasting blood glucose; INR, international normalized ratio; NIHSS, National Institutes of Health Stroke Scale; PLT, platelet; PT, prothrombin time; UA, blood uric acid.

^a^ Conduct *t* test.

### 3.3 Independent risk factors of CMBs

APTT and male gender, which were statistically significant in the above univariate analysis, were analyzed by multifactor logistic regression. It was found that APTT and male gender were independent risk factors for CMBs in patients with ICH (OR 1.100, 95% CI: 1.026–1.180, *p* = .008; OR 2.957, 95% CI: 1.500–5.826, *p* = .002; respectively; as shown in Table 3).

**TABLE 3:** Multivariate logistic regression analysis to demonstrate independent risk factors associated with CMBs

	B	SE	Wald	*p* value	OR	95% CI
APTT (s)	0.095	0.036	7.107	.008	1.100	1.026–1.180
Male	1.084	0.346	9.811	.002	2.957	1.500–5.826

Note

Conduct multifactor logistic regression analysis. Whether CMBs occurred was set as dependent variable, and APTT and male as independent variables, with stepwise forward method was adopted for model selection.

Abbreviations: APTT, activated partial thromboplastin time; CI, confidence interval; CMBs, cerebral microbleeds; OR, odds ratio.

### 3.4 Predictive value of independent risk factors for CMBs

**FIGURE 1:**
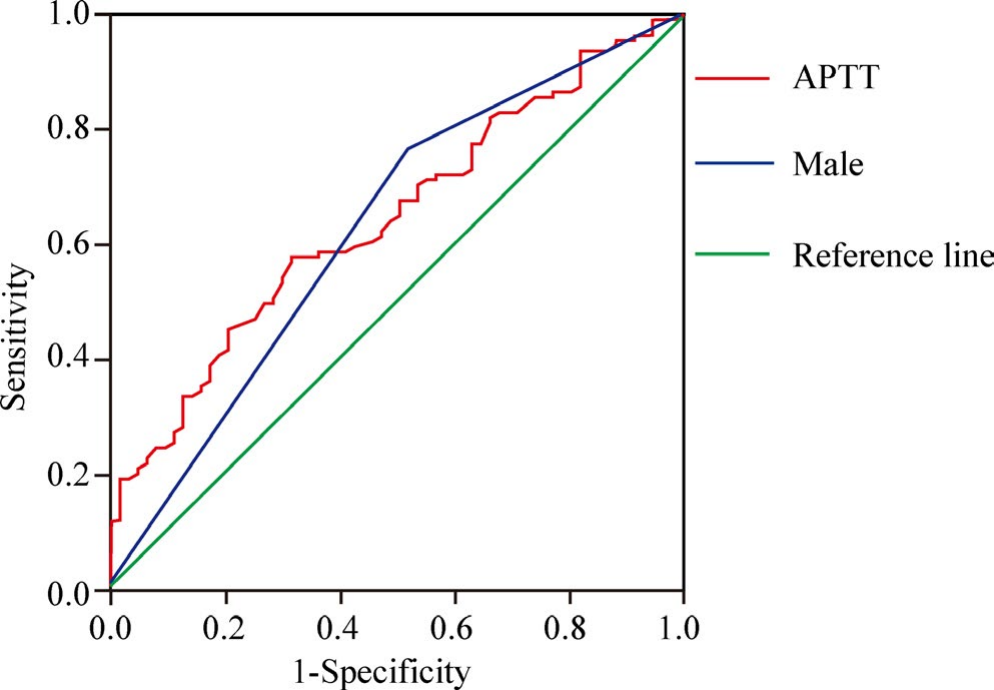
ROC curve for occurrence of CMBs predicted by APTT and male. ROC curve analysis indicated that the area under the curve of APTT and male gender for CMBs in patients with ICH was 0.641 and 0.621, respectively (*p* = .002 and *p* = .008; respectively). APTT, activated partial thromboplastin time; CMBs, cerebral microbleeds; ROC, receiver operating characteristic

To further determine whether APTT and male gender had any independent predictive value, the ROC curve was plotted (as shown in Figure 1). The results demonstrated that the AUC of APTT for CMBs in patients with ICH was 0.641 (*p* = .002), with the sensitivity of 57.7% and the specificity of 68.3%. When Youden's maximum index was 0.26, the APTT value was equal to 30.15 s as the best dividing line. The AUC of male gender for CMBs in patients with ICH was 0.621 (*p* = .008), with the sensitivity of 76.58% and the specificity of 52.38%.

## 4 DISCUSSION

In our study, a comparison between CMBs and non‐CMBs groups found that male was significantly associated with CMBs, and the probability of male patients displaying CMBs was nearly threefold that of female patients. Both Roob et al. (1999) and Jeerakathil et al. (2004) investigated the risk factors for CMBs and found that the occurrence of CMBs was closely related to the male gender. The studies by Sveinbjornsdottir, Sigurdsson, & Aspelund (2008) and Ding, Sigurdsson, & Garcia (2015) revealed that the proportion of male was significantly higher than that of female patients with CMBs. The result of our study was also consistent with the above previous studies.

The study conducted by Lee, Ryu, & Roh (2009) about the coagulation function in 24 patients who developed ICH after warfarin therapy showed that an increase in INR aggravated the occurrence of CMBs. It was reported by Liu et al. (2017) that, for 85 patients combined with atrial fibrillation and ischemic stroke, FBI was an independent risk factor for CMBs (OR 2.16, 95% CI: 1.20–3.90; *p* = .01). The above study conducted by Liu et al. (2017) showed that the values of APTT and TT were not significantly different between CMBs group and non‐CMBs group. However, there are few evidences about the direct relationship between coagulation function and CMBs.

In our study, it also came to the conclusion that coagulation function was associated with CMBs. However, it was inconsistent with the above previous studies that APTT was confirmed to be an independent risk factor for CMBs, but no INR and FBI. Although the predictive valve was low, the AUC of APTT was still with statistical significance, and it might prove that the APTT exists a certain influence on CMBs of patients with ICH on the other side. We speculated there were several reasons for it. First, in the above previous studies, anticoagulant drugs were applied, which would have affected coagulation parameters. However, no any anticoagulant drug was applied in our study. Secondly, the above previous studies were aimed to healthy person or patients with atrial fibrillation and ischemic stroke, but the enrolled patients in our study were with ICH. The patients enrolled were thus different, which might have resulted in differences in the assessed coagulation parameters. In addition, there might have been differences between racial populations. Studies have suggested that the doses required to achieve the same level of anticoagulant intensity between populations of varying racial identities were different (Shen, Yao, Brar, & Chen, 2007). We speculated that the sensitivity of coagulation indicators might indeed vary by racial identity. Our study was performed in Asian patients, as the above study by Lee et al. (2009) was performed in non‐Asian patients; thus, it might lead to the different associations between coagulation function and CMBs. Finally, it was reported that the median coated PLT level in patients with CMBs was significantly reduced (Prodan, Vincent, Padmanabhan, & Dale, 2009), which affected the production of thrombin and the coagulation process. APTT is the main indicator that reflects the state of endogenous coagulation in the body and would change as the state of coagulation changes. Thus, there might be a possible association between APTT and CMBs; however, it was lack of relevant basic research to demonstrate so far.

There were limitations in our study. First, the sample size was relatively small, and only the patients with ICH in the basal ganglia and <30 ml were enrolled. It might lead to a certain selection bias. Secondly, our study mainly illustrated the phenomenon that APTT affected CMBs of patients with ICH independently; however, the relevant mechanism is still not completely clear and targeted study will be needed.

## 5 CONCLUSION

Our study showed that APTT is an independent risk factor for CMBs. Our study revealed that prolonged APTT was an independent risk factor for CMBs in patients with ICH. In clinical work, for ICH with prolonged APTT, MRI‐SWI sequence should be advised for screening CMBs, which can further guide the treatment assessment. However, the further study with enlarged sample and basic study are all needed in the future, in order to demonstrate the mechanism for the association between APTT and CMBs.

## CONFLICTS OF INTEREST

All authors declare to have no conflicts of interest.

## AUTHOR CONTRIBUTION

Dr. Yitao He was in charge of the study design; Dr. Hui Zhang was in charge of manuscript draft; Dr. Jian Deng was responsible for data collection; Dr. Nianlong Sun was in charge of the imageological examination for patients; Dr. Liangyu Zou was responsible for manuscript revision; Nurse Chen Wei was in charge of the blood examination; Dr. Jing Han was responsible for data entry and verification.

## Data Availability

The data of our study will be available via connecting with Dr. Yitao He (corresponding author).
